# Supplementary material for: Inter-Individual Variation in DNA Methylation Patterns across Two Tissues and Leukocytes in Mature Brahman Cattle
Source: Biology (Basel). 2023 Feb 5;12(2):252. doi: 10.3390/biology12020252 (PMC9953534; doi:10.3390/biology12020252)
Supplement: Supplementary file 1 [file biology-12-00252-s001.zip › Supplementary Figures.pdf]

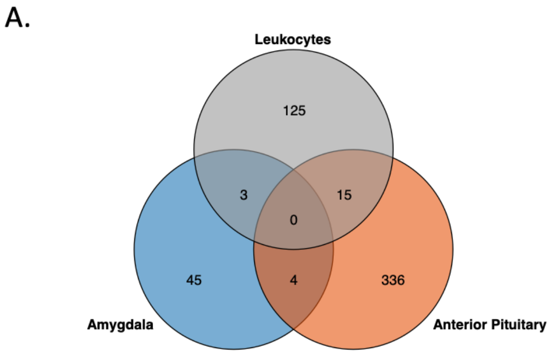

Supplementary Figure S1. Overlap of promoter regions (1000 bp upstream of the transcription start site and 500 downstream from the transcription start site) with high DNA methylation variation across the tissues and leukocytes in the A) Control and B) Prenatally Stressed group.

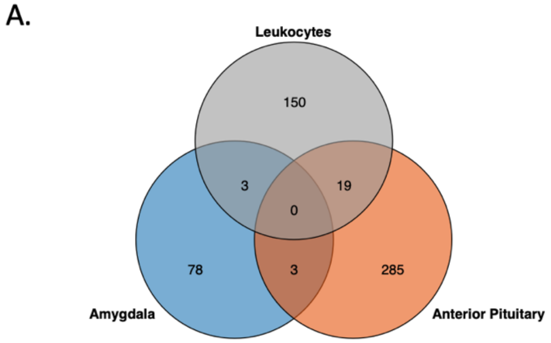

Supplementary Figure S 2. Overlap of cytosine-phosphate-guanine islands with high DNA methylation variation across the tissues and leukocytes in the A) Control and B) Prenatally Stressed group.

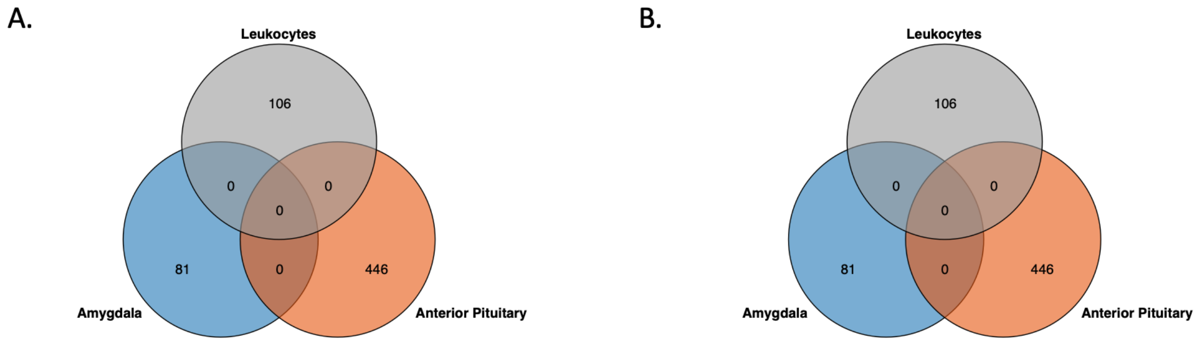

Supplementary Figure S3. Overlap of cytosine-phosphate-guanine shores (2,000 bp upstream and 2,000 bp downstream of cytosine-phosphate-guanine islands) with high DNA methylation variation across the tissues and leukocytes in the A) Control and B) Prenatally Stressed group.

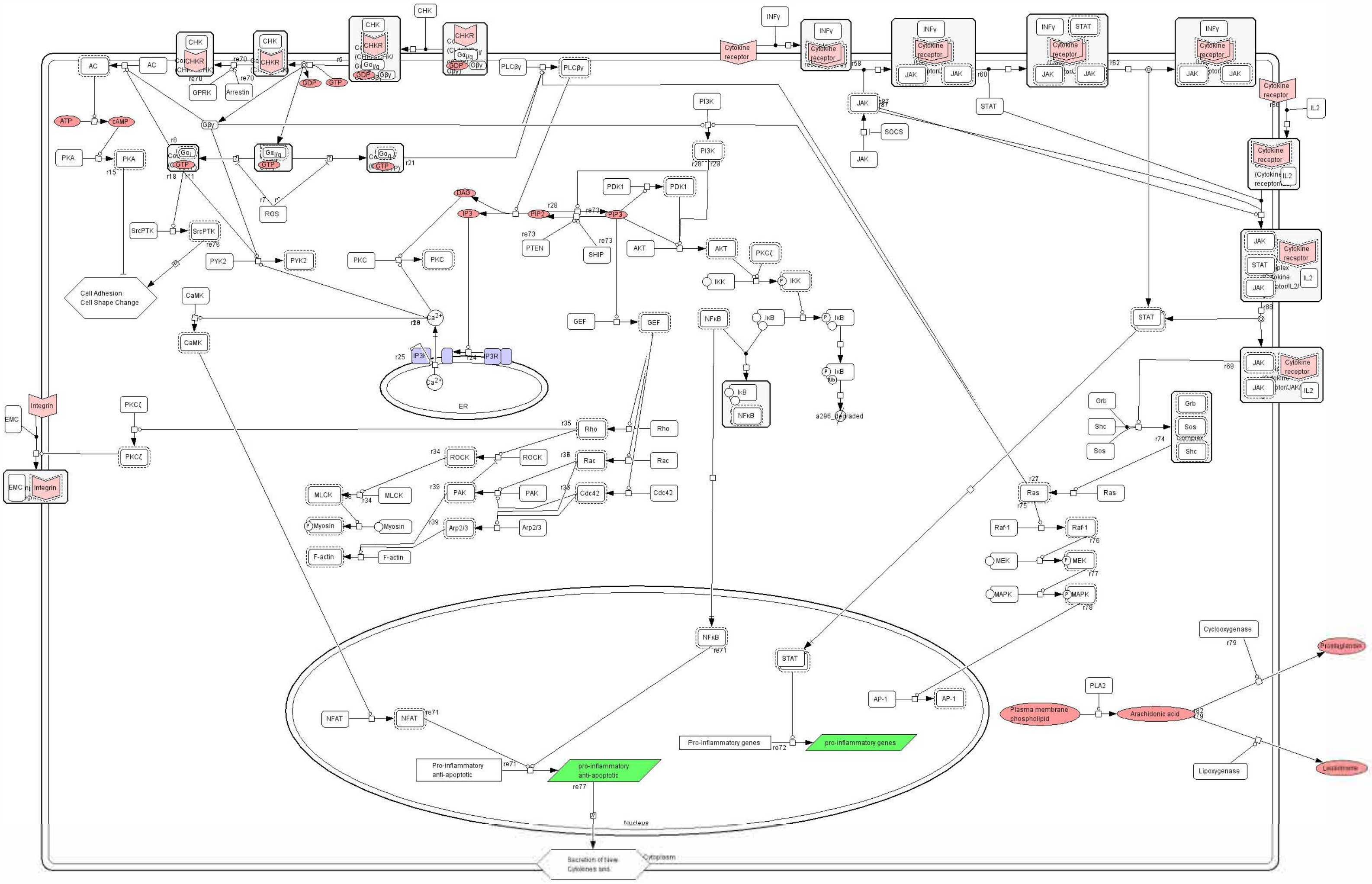

Supplementary Figure S4. Inflammation mediated by chemokine and cytokine signaling pathway
